# Comparison of Fentanyl and Dexmedetomidine as Intrathecal Adjuvants to Spinal Anaesthesia for Abdominal Hysterectomy

**DOI:** 10.31729/jnma.3739

**Published:** 2018-10-31

**Authors:** Binod Gautam, Sushila Tabdar, Ujma Shrestha

**Affiliations:** 1 Department of Anaesthesia and Intensive Care, Kathmandu Medical College, Sinamangal, Kathmandu, Nepal.

**Keywords:** *Abdominal hysterectomy*, *Dexmedetomidine*, *Fentanyl*, *intrathecal adjuvant*, *spinal anaesthesia*, *visceral pain*

## Abstract

**Introduction:**

Spinal anaesthesia, although advantageous for conducting abdominal hysterectomy, is not the first choice amongst surgeons for fear of intra-operative visceral pain. Intrathecal adjuvants may improve quality of spinal anaesthesia. This study aims to compare efficacy of intrathecal Fentanyl and Dexmedetomidine to reduce visceral pain during abdominal hysterectomy performed under spinal anaesthesia.

**Methods:**

Sixty women undergoing abdominal hysterectomy for benign indications were randomly assigned to two equal groups in a double-blind fashion. Fentanyl 25 micrograms in group A or Dexmedetomidine 10 micrograms in group B was co-administered with hyperbaric Bupivacaine 15 milligrams for spinal anesthesia. Surgery through Pfannenstiel incision proceeded once sensory block reached eighth thoracic dermatome. The intra-operative visceral pain was assessed using a five-point scale: none, mild, intermediate, severe, and failed spinal anaesthesia. Duration of analgesia and peri-operative events were studied for 24 hours. Chi-square test, Mann-Whitney U-test and Student's t-test were used for analysis. Level of significance used was P<0.05.

**Results:**

Fifty eight participants completed the study. Demographic variables and sensory block were similar between groups. General anaesthesia was not required in both groups. Significantly greater number of patients in group A required medications for visceral pain with Relative Risk of 2.8 (1.16–6.7). Pruritus and shivering occurred significantly higher in group A. Hypotension was significantly higher in group B. Post-operatively, group B patients showed a significantly longer duration of analgesia.

**Conclusions:**

Dexmedetomidine is better than Fentanyl as an intrathecal adjuvant to spinal anaesthesia in minimizing visceral pain during abdominal hysterectomy and in prolonging postoperative analgesia.

## INTRODUCTION

General anaesthesia (GA) is the gold standard anaesthetic technique for intra-abdominal surgeries. However, it contributes to pain and nausea-vomiting after abdominal hysterectomy (AH).^1, 2^ Spinal anaesthesia (SA) offers numerous advantages over GA, but, visceral pain occurring during uterine surgeries remains a source of anxiety.^3, 4^

Fentanyl, when added to SA, minimizes visceral pain associated with peritoneal traction during uterine surgeries.^5, 6^ Dexmedetomidine, a newer alpha-2 adrenoceptor agonist, is claimed to augment local anaesthetic action in SA.^7, 8^ Intrathecal Fentanyl and Dexmedetomidine are shown to enhance block characteristics and improve quality of SA in various surgical scenarios. But studies comparing their efficacy in reducing visceral pain during AH are not available.

This study aims to compare Fentanyl and Dexmedetomidine as intrathecal adjuvants to SA in women undergoing AH for benign indications. The primary endpoint was the occurrence of intra-operative visceral pain. Assessing the duration of analgesia and peri-operative events for 24 hours comprised the secondary objectives.

## METHODS

After obtaining approval from Institutional Review Committee, a comparative study was conducted from January to September 2018 at the setting of an operating room and post-anaesthesia care unit of a teaching hospital. Informed written consent was taken from participants during pre-anaesthetic checkup performed a day before surgery.

Women admitted to our hospital for undergoing elective abdominal hysterectomy for benign indications were the source of our participants. Consenting women of American Society of Anesthesiologists' physical status 1 or 2, age 30 to 65 years and who understand and speak Nepalese language well were selected for the study. When the principal surgeon was reluctant or was planning for a vertical incision, patients were excluded. Patients with following conditions were also excluded: height<150 cm, weight>100 kg, raised intracranial pressure, severe hypovolemia, moderate to severe stenotic valvular heart disease, bleeding diathesis, spinal anomalies or local infection, allergies to Bupivacaine, Fentanyl or Dexmedetomidine and, patients taking beta-blockers, antiarrhythmics, anticoagulants or anti-depressants.

All the participants were thoroughly informed about the importance of immediate reporting of pain in order to receive analgesics. Specifically, they were instructed to notify any discomfort, anxiety, nausea, and pain during surgery. Numeric Rating Scale (NRS; 0 = no pain and 10 = worst imaginable pain) for grading the intensity of pain was explained. Participants were kept nil per mouth of six hours prior to surgery for solid foods. Premedication included Alprazolam, Metoclopramide, and Ranitidine orally on the night before and on the morning of surgery. Regular medications were allowed as usual.

**Sample size was calculated using the formula**,


$$\text{S}{\text{= z}}^{2}{\text{pq/d}}^{2}$$

Considering hospital prevalence of 1.5% and a confidence interval of 95%, z equals 1.96 and d of 0.05, a total of 23 participants per group would be sufficient. Adjusting 30% for block failure and drop outs, the study was continued till 60 consecutive participants (30 per group) were enrolled using simple randomization. Computer-generated random number list, prepared by the principal investigator, was used for group allocation. It was concealed in serially numbered sealed opaque envelopes that were opened at the time of intervention.

In operating room baseline systolic blood pressure (SBP), heart rate (HR) and respiratory rate (RR) were recorded. Intravenous (IV) access was secured with 18 G cannula to pre-load 10ml/kg Ringer's Lactate and to administer all further medications. The intra-operative fluid infusion was kept at 10 ml/kg/hr.

SA was performed under aseptic conditions in a sitting patient at L3/L4 or L4/L5 intervertebral space using a 27 G pencil-point spinal needle through midline. Total of 3.5 ml of drug solution prepared by the principal investigator in a syringe was handed over to another blinded investigator who injected it intrathecally over thirty seconds; and, 0.1 ml cerebrospinal fluid was aspirated before and after injection to confirm free flow. Both groups received three ml 0.5% hyperbaric Bupivacaine (Bupican heavy^TM^: Claris Injectables Ltd, Ahmedabad, India) for SA. In addition, group A (n= 30) received 25 mcg Fentanyl (Fentyl^TM^: 100 mcg/2 ml; National Healthcare Pvt Ltd, Bara, Nepal) and, group B (n= 30) received 10 mcg Dexmedetomidine (Xamdex^TM^: 100 mcg/ml; Themis Medicare Ltd, Abbott-Manufacturer, Uttarakhand, India) intrathecally. Time of completion of injection was recorded as ‘0’ time and all times were calculated from this point. Patients rested supine immediately in a horizontal operating table. Investigators (second and third) enrolling patients, performing SA, providing care and assessing study outcomes were blinded to the group allocation and so were the participants.

Sensory block level was assessed for loss of cold sensation till attaining peak sensory block. Surgical proceedings with Foley's catheterization initiated once T8 sensory block was achieved. The motor block of lower limbs was assessed according to modified Brom-age Scale (Appendix 1).^9^ Inability to attain T8 sensory block and Bromage 3 motor block bilaterally within 15 minutes was defined as block failure and these patients were excluded from analysis.

Surgery was performed through Pfannenstiel incision. The surgical technique was not standardized, except for AH was performed with extra-fascial technique, and peritonealization of pelvis and anchoring of round ligaments to the vaginal cuff or cervical stump were avoided.

Visceral pain occurring intra-operatively was defined as poorly localized discomfort or dragging, pulling heaviness or unpleasant feeling or pain with or without nausea. Participants were encouraged to self-report such experience and were enquired every five minutes interval during surgery. For analysis, visceral pain outcome, as modified from a previous study, was categorized as following^10^: 1=Excellent, no complaints, 2=Mild discomfort, requiring no analgesic, 3=Intermediate discomfort accompanied by nausea requiring anti-emetic, 4=Severe discomfort accompanied by pain requiring analgesic, and, 5=Failed SA, requiring GA.

The occurrence of nausea was correlated with the decrement of SBP and/or HR and addressed first. Nausea-vomiting occurring otherwise was treated with Ondansetron, Metoclopramide, and Dexamethasone as needed. Fentanyl titrated to a maximum of two mcg/ kg was administered for visceral pain of NRS more than three. Anxiety was addressed with Midazolam (maximum three mg). If not sufficient, GA with increments of Ketamine and Propofol, titrated with vital parameters, were utilized.

HR, SBP, RR, electrocardiography, and Oxygen saturation were monitored continually and recorded at five minutes interval throughout surgery, and according to nursing protocol post-operatively. Hypotension (SBP falling more than 20% from baseline) was treated with increasing fluid infusion rate and Ephedrine. Bradycardia (HR <50/min) was treated with Atropine. Respiratory depression (RR <eight/min) was treated with auditory stimulation and encouragement, Oxygen supplementation and respiratory support as appropriate. Level of sedation was assessed every 15 minutes using Ramsay Sedation Score for patients not receiving IV Midazolam or Fentanyl (Appendix 2).^11^

Peri-operative events including hypotension, bradycardia, respiratory depression, nausea, vomiting, shivering, pruritus, headache and any other were recorded throughout the study period. Duration of surgery, total intra-operative fluid infused, blood loss and hourly urine output were also recorded. Post-operatively, first analgesic was administered once NRS for pain exceeded three. Duration of analgesia was defined as a duration from time ‘0’ to time of first analgesic administration. The total number of analgesic doses administered over 24 hours was recorded when the study ended.

For analysis, the statistical package for social science evaluation version 20 (SPSS Inc; Chicago, IL, USA) was used. Data is expressed as mean (standard deviation) standard error of mean, number (percentage), or median (range). Relative Risk (95% confidence interval) was calculated, and Chi-square test was used for comparing nominal measurements, including the primary outcome measure, the intra-operative visceral pain. Student's independent t-test was used to compare groups for the duration of analgesia and other quantitative variables. For ordinal variables, including the severity of visceral pain, Mann-Whitney U-test was used. Level of significance used was P<0.05.

## RESULTS

**Figure 1.**
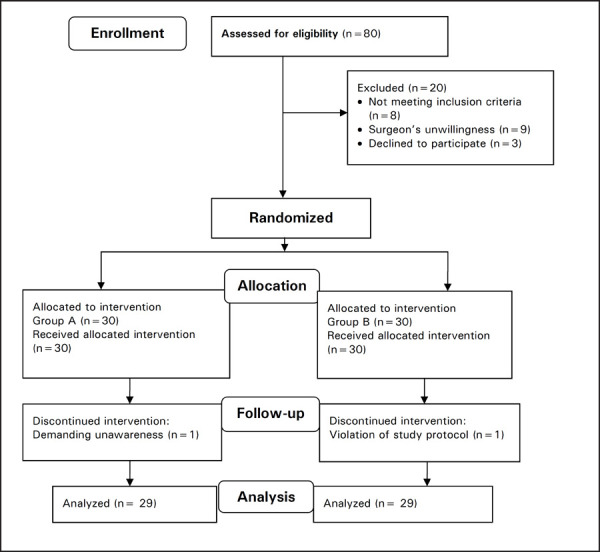
Consort flowchart of the study.

Eighty potentially eligible patients were examined in the study duration. Nine patients were excluded because of principal surgeon's unwillingness and eight patients were excluded because of not meeting inclusion criteria. Whereas three patients denied the consent. Sixty patients were randomized in the study in whom SA was easily performed. In group A, one participant withdrew consent immediately after SA, demanding for unawareness during surgery. Whereas, in Group B, violation of study protocol occurred in one patient due to prophylactic IV anti-emetic administration. Twenty-nine patients in each group were analyzed (Figure 1). There was no block failure and peak sensory block level reached at the median of T4 in both groups (with the range in group A; T6 to T2 and, in group B; T5 to T2).

There was no significant difference between groups in demographic and surgical characteristics (Table 1).

**Table 1.** Demography and surgical profile.

Parameter	Group A (n = 29)	Group B (n=29)	P *
Age (years)	45.86 (6.83) 1.26	45.45 (8.17) 1.51	0.83
Weight (kg)	61.10 (7.72) 1.43	59.97 (7.15) 1.32	0.56
Height (cm)	159.21 (6.7) 1.24	157.41 (5.62) 1.04	0.27
Surgical indication^†^: Uterine myoma / AUB	23 / 6	21 / 8	
Duration of surgery (min)	82.83 (29.57) 5.49	77.48 (18.59) 3.45	0.41
Intra-operative fluid (ml)	1481.03 (249.43) 46.31	1550.00 (317.35) 58.93	0.36
Surgical blood loss (ml)	235.52 (133.80) 24.84	266.38 (157.02) 29.15	0.42
Intra-operative urine output (ml)	102.84 (26.80) 12.42	112.06 (33.46) 18.14	0.40

*Values are mean (standard deviation) standard error of mean*;

* *P values calculated with Student's t-test*;

* *Values are numbers; AUB: abnormal uterine bleeding.*

During surgery, significantly higher number of patients from group A witnessed visceral pain as compared to those from group B (Table 2). Study patients who received Fentanyl as an intrathecal adjuvant to SA had 2.8 times the risk of intra-operative visceral pain compared to patients who received Dexmedetomidine. No patient suffered serious cardio-respiratory events throughout study period except for a significantly higher incidence of intra-operative hypotension in group B, which was, however, easily treated with fluids and vasopressor without affecting outcomes. Sedation score was always three or less in all patients.

**Table 2.** Intra-operative events and medications.

Parameter	Group A (n=29)	Group B (n=29)	Relative Risk (95% CI)	P
Visceral pain	14 (48.3)	5 (17.2)	2.8 (1.16–6.77)	0.022^*^
Hypotension	6 (20.7)	15 (51.7)	0.4 (0.18–0.88)	0.028^*^
Bradycardia	1 (3.4)	5 (17.2)	0.2 (0.02–1.6)	0.19
Shivering	11 (37.9)	3 (10.3)	3.67 (1.14–11.7)	0.029^*^
Pruritus	12 (41.4)	0 (0)	25 (1.5–403.4)	0.023^*^
Midazolam need	13 (44.8)	4 (13.8)	3.25 (1.2–8.79)	0.02^*^
Anti-emetics need	11 (37.9)	3 (10.3)	3.67 (1.14–11.7)	0.029^*^
Fentanyl need	9 (31.0)	2 (6.9)	4.5 (1.06–19.05)	0.041^*^

*Values are number (percentage)*;

*P value calculated using Chi-square test; CI: confidence interval;*

* *Significant statistical difference.*

The severity of intra-operative visceral pain was significantly higher in group A (Table 3).

**Table 3.** Severity of visceral pain.

Visceral pain grade^*^	Group A (n=29)	Group B (n=29)
1=No complaints	15 (51.7)	24 (82.7)
2=Mild discomfort	2 (6.9)	2 (6.9)
3=Intermediate discomfort	3 (10.3)	1 (3.4)
4=Severe discomfort	9 (31.0)	2 (6.9)
5=Requirement for GA	Nil	Nil

*Values are number (percentage)*;

* *Significant statistical difference with Mann-Whitney U-test (P value 0.015).*

Duration of analgesia was significantly prolonged in group B; and, number of analgesic doses consumed over 24 hours in this group was significantly less as compared to group A (Table 4).

**Table 4.** Duration of analgesia and postoperative events.

Parameter	Group A (n = 29)	Group B (n=29)	P
Duration of analgesia^*^	192.52 (28.05) 5.21	318.17 (57.23) 10.62	0.000
Total analgesic doses^*^	5.97 (1.76) 0.327	3.62 (0.67) 0.126	0.000
Nausea^†^	8 (27.6)	4 (13.8)	0.331

* *Values are mean (standard deviation) standard error of mean, and P value calculated with Student's t-test;*

† *Values are number (percentage) and P value calculated with Chi-square test.*

Surgical complications were not observed and no patient required the blood transfusion. One patient from group A developed post-dural puncture headache at 22 hours of surgery and was effectively managed conservatively. Non-specific headache was treated in one and two patients in group A and group B respectively. Two patients in group A received H_2_ blockers for epigastric pain.

## DISCUSSION

The results indicate that Dexmedetomidine 10 mcg when added to 15 mg hyperbaric Bupivacaine in SA, significantly reduces occurrence and severity of visceral pain during AH as compared to Fentanyl 25 mcg. In addition, Dexmedetomidine in comparison to Fentanyl significantly prolongs duration of analgesia and reduces consumption of analgesics.

Dexmedetomidine, a highly selective alpha-2 agonist, has been the focus of interest for its analgesic, sympatholytic and hemodynamic stabilizing properties. Applied intrathecally it interrupts pain transmission by the depressing release of pro-nociceptive transmitters, substance P and glutamate from pre-synaptic C-fibers and by hyperpolarizing post-synaptic dorsal horn neurons in spinal cord.^12^ Intrathecal alpha-2 agonists have been claimed to reduce both somatic and visceral pain.^13, 14^ Surgeries associated with visceral handling and mesenteric traction might be well tolerable for awake patients. Intrathecal dose of 10 mcg Dexmedetomidine is shown to provide optimal favorable effects on SA characteristics without undue risks.^8, 15^

Fentanyl is a mu-receptor agonist opioid. In the spinal cord, it binds with opioid receptors at the dorsal horns, and may also spread rostrally for mediating analgesia. Being lipophilic, Fentanyl, possesses more rapid onset and recovery characteristics. Reduced need for analgesics and anti-emetics reflect its effectiveness against visceral pain when added to SA for cesarean section.^5, 16^ In non-obstetric patients, 10 to 25 mcg is the best risk-benefit dose range.^6^

Because several clinical studies have shown that intrathecal Dexmedetomidine prolongs and improves quality of postoperative analgesia, we were interested to find if it could reduce intra-operative visceral pain. We aimed to compare it with Fentanyl to determine which one is superior in improving the quality of SA during AH. The absence of a control group may be thought of as a limitation of our study. But, as there is higher association between SA with sole local anaesthetic and visceral pain during uterine surgeries, the absence of control group is well justified.

Both agents prevented the conversion of anaesthetic technique to GA in our study. However, occurrence of visceral pain was significantly higher in group A (48vs17%) with the Relative Risk of 2.8. Visceral pain during cesarean section performed under SA has been reported to be as high as 50 to 70%.^3, 4^ No such data is available for AH. As the occurrence of anxiety, nausea, and pain and the need for their treatment were significantly lower in group B, we could conclude that intrathecal Dexmedetomidine minimizes visceral pain and results in better quality of surgical anaesthesia. Intrathecal Dexmedetomidine is shown in experiments to increase visceromotor thresholds in a dose-dependent manner.^13^ And, Yohimbine, an alpha-2 antagonist, could reverse those effects, proving visceral anti-nociceptive effects of Dexmedetomidine. But, clinical applications in its support have been sparse. Gupta R et al compared intrathecal Fentanyl and Dexmedetomidine but reported no difference in visceral pain.^17^ However, surgery was not specified, methods to assess visceral pain were not mentioned and Dexmedetomidine dose was smaller in their study.

Our study has shown that addition of Dexmedetomidine to spinal Bupivacaine significantly prolongs the duration of analgesia as compared to Fentanyl and reduces postoperative analgesic requirement. The results coincide with findings of other authors.^17–19^ Additive and/or synergistic action of Dexmedetomidine with local anaesthetic explains the findings. Whereas, postoperative analgesic requirement has not been shown to decrease with intrathecal Fentanyl, owing to its shorter duration of action.^5^ Mohamed et al have also shown that intrathecal Fentanyl did not add to the duration of analgesia provided by Dexmedetomidine.^19^

The incidence of hypotension in Dexmedetomidine group in our study was significantly higher (52vs21%). Increased risk of hypotension with intrathecal Dexmedetomidine has not yet been established.^20^ A study reported higher requirement of vasopressor for hypotension, although without a statistical significance when five mcg Dexmedetomidine with 12.5 mg Bupivacaine was studied.^17^ This could also emphasize the dose-dependent effects of intrathecal Dexmedetomidine and Bupivacaine. As there was no difference between groups with regards to demography, sensory block and possible confounding variables in our study (Table 1), we could assume that Dexmedetomidine's sympatholytic property, with decrease in plasma catecholamine levels could intensify the SA-induced hypotension. On the other hand, Fentanyl is believed not to increase sympathetic block of SA.^16^ To define hypotension, a moderately lower threshold of 20% decrement in SBP was chosen in our study in order to avoid confusion of its associated nausea with that from visceral pain; and, this could have been contributory. Finally, a relatively smaller sample size of our study, which was not adequately powered for differentiating hypotension might have been responsible.

Intrathecal Dexmedetomidine and bradycardia are shown to be associated.^20^ It is emphasized by our result (17vs3%), even though it did not reach statistical significance. Pruritus was observed in 41% of patients from Fentanyl group. This is reported previously with incidence reaching up to 60%.^5, 16, 19^ Occurrence of shivering was significantly low in Dexmedetomidine group. It is related to Dexmedetomidine's potential to decrease shivering threshold.^21, 22^

SA favors post-surgical recovery, postoperative symptoms, long-term outcomes and costs as compared to GA.^1, 23, 24^ It is associated with reduced risk of chronic pain after hysterectomy.^25^ This must have resulted from effective early post-operative pain control. Even then, apparently many have been reluctant to accept and adopt the routine use of SA for AH. Apart from the fear of intra-operative visceral pain, there are several possible reasons for this, including lack of collaboration in the surgical team and lack of awareness and failure to accept evidence-based data.

Some of the eligible and eager patients were excluded from our study due to unwillingness of principal surgeon. This denotes a source of potential bias and may represent a major limitation of our study. Manifestation of visceral pain is vague and we have to rely on patient's subjective expression. And, as there are no established scales for its assessment in current use, we had a modification from a previous study to match the usual clinical practice in our set up. To address the potential imprecision of the scale, the category of the patient's visceral pain was recorded from the anaesthetic chart only after the anaesthetic management was completed. Lack of evaluation of patients' satisfaction, surgeons' difficulty, and cost-effectiveness and usefulness of the technique in AH for malignancies represent added limitations in our efforts, which warrants further studies.

Anaesthetic techniques have improved drastically over the last two decades. Many drug regimens have been suggested to eliminate anxiety during regional anaesthesia. Most importantly, prevention of pain reduces anxiety resulting in increased patients' compliance and satisfaction. Our study was one of the first of its kind to assess visceral pain occurring during AH in awake patients. And, the finding that intrathecal Dexmedetomidine improves the quality of SA with no remarkable adverse event might further increase the acceptance of this very popular technique not only for AH but for other intra-abdominal surgeries too.

## CONCLUSIONS

We conclude that the incidence and severity of intraoperative visceral pain was reduced markedly, but was not eliminated when an intrathecal Dexmedetomidine was compared with intrathecal Fentanyl during abdominal hysterectomy performed under spinal anaesthesia. But the pain-free postoperative period was prolonged and 24 hours analgesic consumption was also reduced with Dexmedetomidine. As provision of opioid-free analgesic regimen is rational considering relevant evidence of opioids as potent emetic agents with other undesirable effects including pruritus and possible respiratory depression, Dexmedetomidine seems to be the better alternative as an intrathecal adjuvant to Fentanyl, especially when surgery is anticipated to be prolonged and associated with peritoneal traction and visceral stimulation.
